# Assessment of the efficiency of language interpreter services in a busy surgical and procedural practice

**DOI:** 10.1186/s12913-017-2425-7

**Published:** 2017-07-04

**Authors:** Christopher M. Burkle, Kathleen A. Anderson, YaPa Xiong, Andrea E. Guerra, Daniel A. Tschida-Reuter

**Affiliations:** 1 Department of Anesthesiology and Perioperative Medicine, Mayo Clinic, 200 First Street SW, Rochester, MN 55905 USA; 2 Clinical Ethics Committee, Mayo Clinic, 200 First Street SW, Rochester, MN 55905 USA; 3 Language Services Department, Mayo Clinic, 200 First Street SW, Rochester, MN 55905 USA

**Keywords:** Language services, Limited English proficiency, Surgery

## Abstract

**Background:**

Surgical and procedural patient care settings require efficient patient flow. The primary goal of this study was to assess use and efficiency of language services for our limited English proficiency (LEP) patients undergoing surgical and outpatient procedures.

**Methods:**

Patient language services needs were recorded from our operating room and procedural locations over a two and a half month period in 2016. Time from in-person interpreter request to arrival was recorded. Frequency of language service modality used and reason for telephone and professional video remote interpreting (VRI) rather than in person professional services was queried.

**Results:**

Mean time from in-person interpreter request until arrival was 19 min. Variation was high. No cases were cancelled due to lack of available interpretive services and no LEP patient underwent a procedure without requested interpretative service assistance.

**Conclusions:**

Time for in person professional interpreter assistance was short but highly variable. Access to telephone interpretive services and VRI services ensured assistance when in person interpreters were immediately unavailable.

With the numbers of LEP patients increasing over time along with any new mandates for providing language assistance, the stress on hospital patient service units and the financial implications for many health care facilities will likely continue as challenges.

## Background

It is estimated that 24 million people in the United States have limited English proficiency (LEP) [1]. These individuals may have difficulty communicating with health care providers resulting in compromised patient safety and overall poorer health outcomes [1–5]. However, assistance from professional interpreters has been shown to improve health care outcomes and satisfaction among LEP patients [4, 5].

All health care organizations in the United States that receive federal funds are obligated to make available language services to LEP patients under Title VI of the Civil Rights Act of 1964 and subsequent regulations through Section 504 of the Rehab Act of 1973 [2, 5]. More recently, the final rules released on language services under the Section 1557 of the Affordable Care Act of 2010 by the Department of Health and Human Services Office of Civil Rights strengthens and clarifies the obligations required to not only provide language services but also to ensure that they are meaningful [1, 6]. In addition, many states now require some form of language assistance for LEP patients [7].

The National Standards for Culturally and Linguistically Appropriate Services in Health Care (CLAS standards) were developed in 2001 to help assess provider compliance with federal mandates for language services under Title VI of the Civil Rights Act of 1964 [2, 8, 9]. In 2013, these standards were enhanced by the Department of Health and Human Services to better reflect the growing diversity that had occurred in the United States over the decade following the original draft of the CLAS standards. The enhanced standards under the theme titled “communication and language assistance” include:

- Offering communication and language assistance
- Informing individuals of the availability of language assistance
- Ensuring the competence of individuals providing language assistance
- Providing easy-to-understand materials and signage [8, 9]

Despite federal and state mandates, a significant number of hospitals and clinics have still not taken appropriate measures to help ensure adequate communication of health related matters to LEP patients [1, 7, 10]. While the CLAS Standards are not statutory or regulatory requirements per se, failure by hospitals to provide adequate language services to LEP patients has resulted in investigations for violations of Title VI of the Civil Rights Act of 1964 [7, 8, 10].

Our busy hospital and clinic setting is part of a private, not for profit, quaternary health care institution in the United States serving patients from all 50 States as well as a large number of international patients. As such, the need for a comprehensive language service effort to meet the needs of LEP patients is required. However our ability to provide these services to LEP patients in a manner so as to not cause delays to the procedural schedule had been voiced as a possible concern. Therefore, the primary goal of this observational study was to assess the use and efficiency of language services made available to our LEP patients undergoing surgical and outpatient procedures at our facility. As these surgical and procedural types of patient care settings require continued patient flow so as to not delay the time sensitive daily schedule, they were considered the best means of assessing any concerns for the efficacy and efficiency of the language services system in place at our institution.

Our language services department provides assistance to LEP patients through

- the use of either in person professional interpreters
- telephone interpretive services,
- professional video remote interpreting (VRI) services.

The secondary goal of this study was to determine how often each of these particular language service modalities was used by patients and why telephone and VRI use rather than in person professional services were chosen in certain cases. Given that financial burden is often highlighted as a limitation in providing language assistance services to LEP patients, we also explored the cost to our institution associated with providing interpretive services over the study period.

## Methods

Following Mayo Clinic Institutional Review Board approval, the needs for patient language services were recorded from our operating room and procedural locations over a two and a half month period (June 13, 2016 and September 30, 2016).

Information obtained for each encounter with an LEP patient included whether assistance for language needs was requested and the primary language of each patient using this service. If an interpreter was requested, the type of language service provided (in person professional interpreters, telephone interpretive services, professional videoconference services) was also recorded. When an in person interpreter was used, the time from initial request to arrival of that interpreter to the pre-anesthesia or pre-procedural areas was documented. Additionally, the reason for using the telephone interpretive services or professional VRI services rather than an in person interpreter was also recorded.

Costs for providing interpretive services for 2016, the year in which the study took place, were determined through analysis departmental accounting datasheets.

Descriptive statistical analyses were performed.

## Results

Over the study period, 354 LEP patients visited our surgical and outpatient procedural areas. Information for 36 patients was missing leaving 318 records available for analysis. Among those 318 LEP patient records, 308 patients (97%) chose the assistance of the hospital language services available to them while 10 patients (3%) opted for assistance from either a family member or other acquaintance. (Table 1) Over the study period, LEP patients with a total of 18 unique primary languages visited our surgical and procedural centers. The five most common primary languages were Arabic, Spanish, Somali, Chinese Mandarin, and Vietnamese with the remainder making up less than 2% of the total languages among LEP patients. Overwhelmingly, in person language interpreters were used most commonly for providing interpretive services. At the time of our study, our institution offered only telephone or professional VRI language services for LEP patients with a primary language of Korean (1.9%), Hmong (0.9%), or Farsi (0.6%). Since completion of our study, an in-person interpreter has now been hired on staff to provide in-person interpretive services to our LEP Hmong patient population.

**Table 1:** Interpreter service use

Language services assistance used (318 patients)	
Yes	308 (97%)
No	10 (3%)
Language types (most common)	
Arabic	(125) 39%
Spanish	(57) 18%
Somali	(43) 14%
Chinese Mandarin	(15) 5%
Vietnamese	(8) 3%
Type of interpreter services used (305 of 308 total patients with information available)	
In person	241 (79%)
Telephone	55 (18%)
VRI	9 (3%)

The mean time from calling for an in-person interpreter until their arrival at the bedside of the patient was 19 min. The standard deviation was 17.5 min with a range from 0 to 100 min. The median and mode were 17 min and 0 min, respectively. Those cases in which the time to wait for an in person interpreter was 0 min reflected the fact that the interpreter had arrived with the patient. We separately calculated a median wait time of 23 min in those cases where an in person interpreter was called by the operating room or procedural area staff.

Reasons for choosing to use either telephone interpretive services or professional VRI services rather than in person interpreters were available for 38 of the 67 patients in which in person interpreters were not used. The three most common reasons for use of telephone interpretive services or professional VRI services included patients with primary languages where in-person interpreters were not on staff (14 patient events), the wait times for in person interpreters were too long (13 patient events), and in person interpreters were known to be unavailable (10 patient events) at the time of the initial request. In one instance, the initial choice was to use VRI yet a VRI interpreter was unavailable so an in person interpreter was subsequently contacted to provide the services needed. In only two cases patients refused the use of interpretive services available to them. No cases were cancelled due to lack of available interpretive services. Furthermore, in no instance did an LEP patient undergo a procedure without some form of interpretative service assistance being used.

Total institutional language departmental costs for providing interpretive services in the year 2016 were calculated to be $5,847,000. This included both $4,290,000 for salary and benefits of in-person interpreters and $1,528,000 for VRI and telephone vendor services.

## Discussion

In our private not for profit hospital, 362 LEP patients visited our surgical and procedural areas involving anesthesia services. In July 2010, The Office of the Inspector General (OIG) under the Department of Health and Human Services reported on data from a 2009 voluntary survey of 140 randomly selected Medicare providers asking them to respond to four of 14 CLAS standards considered reflective of what language access services should be offered by providers [11]. CLAS standard 4 states that “health care organizations must offer and provide language assistance, including bilingual staff and interpretive services, at no cost to each patient/consumer with limited English proficiency at all points of contact, in a timely manner during all hours of operation.” [11] In the OIG report, only 64% of hospitals surveyed stated they consistently met this standard [11]. In a separate study published in 2010, Diamond and colleagues reported that a majority of providers who responded their survey claimed the ability to providing language services to patients 24 h a day, 7 days a week [2]. In a more a recent report analyzing nationwide data of over 4500 hospitals found that over 30% of hospitals failed to offer language services [1]. When stratified according to hospital type, Schiaffino and colleagues found that private not for profit hospitals like ours provided LEP services more commonly than either private for profit or government hospitals. As pointed out by the authors, given that the number of private for profit hospitals is increasing across the United States, an increasing lack of language services for LEP patients may follow [1].

One of the goals of our study was to determine the length of time required for in person interpreter services to arrive, and then as a consequence, whether extended wait times may have resulted in cases either being cancelled or proceeding without use of interpretive services. The mean wait time for in person interpreters was 19 min but the range extended out to 100 min. No patients were reported to have undergone a procedure without the aid of language services because of an extended wait time for an in person interpreter. In those cases where an in person interpreter was either initially known to be unavailable or when wait times were such that possible delays in cases might occur, either telephone interpretive services or professional videoconference services were used instead. Diamond and colleagues reported that 78% of respondents to their survey of emergency departments stated the ability to provide language services within 15 min of patient arrival for their most common language with less than half (48%) having that ability for their third most common language need [2]. They reported that the most common reasons for delay were specific language needed, time of day, a limited number of available interpreters, location of the interpreter at the time of the request, urgency of the need, and clinical location of the needed language services. While the clinical settings we analyzed did not require 15 min response times, extended periods of wait time would risk case delay. Timely access to telephone interpretive services or professional VRI services resulted in no cancelled cases or patients undergoing procedures without having been provided some form of interpretive services. Similarly, contracting for these services may assist other hospitals and clinics in situations when a dependence on use of in person interpreters is not feasible.

Language assistance for LEP patients can take many forms including use multilingual physicians and other health care staff, in person professional interpreters, telephone interpretive services, professional VRI services, written translation of forms and educational materials, and use of informal interpreters such as patient family members and friends [2]. Despite access to either in person professional interpreters, telephone interpretive services, or professional VRI services, 10 patients (3%) in our study refused the assistance of hospital based interpretive services with either family members or other acquaintances providing interpreter language assistance instead. However, this percentage is significantly lower than the almost 2.5 fold increase (24% vs. 59%) between 2002 and 2008 reported by Ginde et al. when evaluating language assistance trends among patients visiting emergency departments in Boston, following implementation of mandatory interpreter legislation in Massachusetts [12]. The importance of using professional interpreters either alone or in combination with a family members or friend is well established. In a systematic review by Karliner et al. published in 2007, the use of professional interpreters versus ad hoc interpreters (defined as family members or bilingual hospital staff) was found to improve the quality of care received by LEP patients [4]. Following an analysis of videotaped patient-physician encounters with either a professional or family member assisting with interpretive services, Rosenberg and colleagues found that professional interpreters largely transferred information directly between the doctor and the patient while family members acting as interpreters were more likely to speak as themselves [13]. They concluded that while a family member may serve a beneficial role as an advocate for the patient, use of a professional interpreter is still needed to ensure that information is accurately delivered between the patient and the physician [13].

Our institution has a written policy that strongly discourages the use of family members as interpreters except in “life threatening situation(s)” when interpretive services are not available in a timely manner or when a patient refuses such services. In those situations, medical providers may request the presence of institutional provided interpretive services to ensure the accuracy of informational exchange. Diamond et al. found that 62% of hospitals in their 2010 survey reported patient family members or friends being used as interpreters for LEP patients [2]. Seventy percent of these hospitals had policies in place restricting this practice to situations including where patients refused hospital available resources, emergency scenarios, if a waiver was signed by the patient, or at the physician’s discretion [2]. Some of the policies disallowed patient or family members to be used in particular clinical situations such as discussions about invasive procedures, treatment planning and informed consent [2]. It should also be noted that under Section 1557 of the Affordable Care Act of 2010, use of unqualified interpreters is prohibited [6].

The total cost for providing interpretive services in 2016 at our medical campus was $5,847,000. While only a fraction of this cost was directly attributable to patients visiting the surgical and procedural practice areas we surveyed in our study, the availability of in person interpreters to meet the needs of all our patients requires a total of 43 full time employee equivalent interpreters. In addition to salary and benefit costs for in person interpreters ($4,290,000 at our medical campus in 2016), there is the cost of contracted services for telephone interpretive services and professional VRI services ($1,528,000 at our medical campus in 2016). The State of Minnesota is one of 13 States that offer some amount of reimbursement to health care providers for costs attributable to offering language services to LEP patients [7]. While the cost of providing interpretive services can be high, limited reimbursement for hospitals and other providers exists [7]. Medicare does not reimburse for language access services. State Medicaid Programs and Children’s Health Insurance Programs (SCHIP) can collect Federal matching funds to reimburse providers in their state for the costs of interpretive services [7, 11]. Our medical institution does not charge patients or insurers for the cost of providing interpretive services however they may seek reimbursement from the State of Minnesota for those patients receiving medical assistance.

In a 2010 report by the Office of the Inspector General of the United States, 27% of providers reported that cost of offering language services was an obstacle [11]. Only 3% of the providers in the survey received reimbursement through State or local government, or Medicaid systems [11]. Forty-five percent of those surveyed stated that additional assistance, including financial assistance, would be useful in improving compliance with offering language services to LEP patients. Despite these cost concerns, very few of those surveyed offered information as to their costs in providing language services. Annual costs for providing language services ranged from $50 to $779,494 while per LEP patient costs varied from $0.33 to $1500 [11]. In a 2006 State of Connecticut report, it was estimated that it would cost approximately $4.7 million to provide language services to 22,353 LEP patients covered under the state Medicaid program and using 4.6% of total Medicaid services [14]. A 2008 report on language access services in critical access hospitals in rural Minnesota found that 25% of hospitals reported facing significant financial burdens in providing these services [7]. In a survey prepared by Diamond and colleagues, cost and lack of insurance reimbursement was the most common reason identified by hospitals for noncompliance in offering language services [2]. Interestingly however, there was no difference in reported compliance rates among those hospitals in States that offer some amount of reimbursement through their Medicaid programs. While Medicaid and SCHIP interpretive services can be covered, a gap still exists in reimbursement for services provided to Medicare and other patient service reimbursement populations [5, 7]. With increasing numbers of LEP patients over time along with any new mandates for providing language assistance, the financial implications for many health care facilities will likely continue to be a challenge.

## Conclusion

In summary, access to in person professional interpreters, telephone interpretive services, and professional VRI services caused none of our LEP patients to be cancelled or delayed for their procedures. However, while the mean time for assistance from an in person professional interpreter was reasonably short, there remained instances of extended wait times. Access to both telephone interpretive services and professional VRI services helped to ensure that LEP patients received the assistance they needed when in person interpreters were unavailable.

While the patient population served by our tertiary care center may not represent the majority of hospitals in the United States, our findings emphasize the administrative and financial burdens that health care entities have in providing language services. These challenges will likely continue as the population of LEP patients grows in the future. While our present study was primarily designed to assess the efficiency of interpretive services, it will be important for future studies to determine the quality of these different interpretive service modalities in meeting specific patient and practitioner needs, informed consent as just one example, in our busy practice.

## Abbreviations

LEP: Limited English proficiency
VRI: Professional video remote interpreting (VRI) services
